# Phenomenological Qualitative Methods Applied to the Analysis of Cross-Cultural Experience in Novel Educational Social Contexts

**DOI:** 10.3389/fpsyg.2022.785134

**Published:** 2022-04-25

**Authors:** Ahmed Ali Alhazmi, Angelica Kaufmann

**Affiliations:** ^1^Education Department, Jazan University, Jazan, Saudi Arabia; ^2^Cognition in Action Unit, PhiLab, University of Milan, Milan, Italy

**Keywords:** qualitative research, phenomenology, cross-cultural analysis, educational research, experience

## Abstract

The qualitative method of phenomenology provides a theoretical tool for educational research as it allows researchers to engage in flexible activities that can describe and help to understand complex phenomena, such as various aspects of human social experience. This article explains how to apply the framework of phenomenological qualitative analysis to educational research. The discussion within this article is relevant to those researchers interested in doing cross-cultural qualitative research and in adapting phenomenological investigations to understand students’ cross-cultural lived experiences in different social educational contexts.

## Introduction: The Qualitative Method in Educational Research

Many scholars in phenomenology hold the view that human beings extract meaning from the world through personal experience (Husserl, 1931; Hycner, 1985; Koopmans, 2015; Hourigan and Edgar, 2020; Gasparyan, 2021). Investigating the experience of individuals is a highly complex phenomenon (Jarvis, 1987): annotating and clarifying human experience can be a challenging task not only because of the complexity of human nature, but also because an individual’s experience is a multidimensional phenomenon, that is, psychologically oriented, culturally driven, and socially structured. Hence, much uncertainty and ambiguity are surrounding the description and exploration of an individual’s experience. Such uncertainty is due to the multidimensional aspects that constitute and form an individual’s experiences, including ongoing and “mediated” behaviour (Karpov and Haywood, 1998), feelings, and cognition. In all these respects, the complexity of experience becomes especially evident in certain investigative contexts such as the one we decided to explore, that is the study of the cross-cultural interactions of individuals who experience a transition from their own cultural and educational social context to a different one. In this article, it is argued that a hybrid phenomenological qualitative approach that, as shall be illustrated, brings together aspects of descriptive phenomenology, and aspects of interpretative phenomenology (Moustakas, 1994; Lopez and Willis, 2004), could assist researchers in navigating through the complexity of cross-cultural experiences encountered by individuals in novel social educational contexts. Descriptive phenomenology was derived mainly from the philosophical work of Husserl and particularly from the idea of transcendental phenomenology (Moustakas, 1994; Lopez and Willis, 2004; Giorgi, 2010). In contrast, an interpretive phenomenological methodology was derived from the works of scholars like Merleau-Ponty (1962), Gadamer (2000), and Gadamer and Linge (2008). These two approaches overlap in the research methods and activities and are deployed to assist the research by promoting engagement with responsive and improvised activities rather than with mechanical procedures. The general qualitative methodology of social science research has shaped phenomenology as a methodological approach just as reliable as quantitative and experimental methods, as recently discussed by Høffding et al. (2022), who stressed the advantages of phenomenology in qualitative research (see also Zahavi, 2019a,b). Since we are interested in cross-cultural experiences, in the past, for example, we used such phenomenological qualitative type of investigation to find out what it is like for Saudi international students to transition from a gender-segregated society to a mixed-gender environment while studying and living as international students (Alhazmi and Nyland, 2013, 2015). We were interested in further understanding the phenomenon of transitioning itself rather than collecting students’ opinions and perspectives about the experience of transitioning. The investigation was conducted to capture and describe essential aspects of the participants’ experience, to understand the experience encountered by students in their novel social educational context. Besides this specific study case, the same hybrid methodology, as shall be suggested, may be applied to the study of similar types of social environments and groups. We refer to our past work on cross-cultural transitioning experience to help the reader translate how the phenomenological qualitative methodology can be applied in relatable scenarios in educational research.

As Giorgi (1985), Van Manen (1990), Moustakas (1994), and other phenomenologists have stated, interviewing individuals who experience specific phenomena is the foundation source that phenomenological investigation relies on to understand the phenomenon. Accordingly, aspects that are core to the interviews are the following: (1) general attributes of the conducted interviews, (2) criteria of selection for potential participants, (3) ethical considerations of dealing with human participants, and (4) the interviewing procedures and some examples (these will be presented in section “Practising Phenomenology: Methods and Activities”).

To design a phenomenologically based qualitative investigation, we suggest considering three aspects: (1) the aim of the investigation; (2) the philosophical assumptions about the sought knowledge; and (3) the investigative strategies. These three aspects of the investigation shall be approached while keeping in mind the two following rationals: (1) looking for essence and (2) flexible methods and activities.

1. The researcher’s aim is that of identifying the essential and invariant structure (i.e., *the essence*) of the lived experience as this is described by the participants (Moustakas, 1994; Crotty, 1998; Cresswell, 2008). This allows the researcher to ‘return to the concrete aspect of the experience’ (Moustakas, 1994, p. 26) by offering a systematic attempt to present the experience as it appears in consciousness (Polkinghorne, 1989) and to focus on the importance of the individuals and their respective views about the lived experience (Lodico et al., 2006). It is essential to keep this aim (i.e., *identifying the essence of lived experience*) in mind when conducting a phenomenological qualitative investigation as this is the core aim of phenomenology. According to Finlay (2006, 2008), exploring and understanding the essential structure and themes of the lived experience encountered by individuals is critical. Researchers adopting these perspectives ‘borrow’ the participants’ experience and their reflections on their experience to get a deeper understanding and to grasp the deeper meaning of the investigated experience (Van Manen, 1990, p. 62). This is what Finlay (2006, 2008) calls ‘dancing’ between two approaches, and it is also the approach that we endorse. As pointed out by Høffding and Martiny (2016), in this explorative process the interviewer needs to understand the relation between the interviewee’s experience and their description of it, since the interview constitutes a second person perspective in which one directly encounters another subjectivity and shall not elicit closed answers such as “yes” or “no” (see section “Attributes of the Conducted Interviews”). This feature is useful when exploring an experience that has not been sufficiently explored and discussed.
2. The suggested phenomenological qualitative approach offers a strategy that ‘sharpen the level on ongoing practices in phenomenologically inspired qualitative research’ (Giorgi, 2006a, p. 306). Methods and activities for data collection are flexible, and the analysis is designed to be aligned with the theoretical and philosophical assumptions underlying qualitative research. The present strategy allows a researcher to dialogue with both the participants and the data to produce a multi-layered description of the experience. This feature is academically important in terms of conducting a rigorous qualitative study that provides trustworthy knowledge (Crotty, 1998; Denzin and Lincoln, 2003; Creswell, 2007, 2009; Bryman, 2008).

The three core aspects of the investigation (1) *the aim of the investigation;* (2) *the philosophical assumptions about the sought knowledge; and* (3) *the investigative strategies, informed by these two rationals*, are essential to developing a phenomenologically oriented qualitative method to examine the lived experience and for identifying its essence.

## Aim and Method

Thinking about the actual object of our investigation, that is, the lived experience of individuals is an essential aspect of phenomenological qualitative research. The researchers need to identify their aim very carefully by focusing on the lived experience of the subject being interviewed and on the structure of such experience rather than on the opinion of the participants about the experience.

In our previous studies, we were interested in describing the cross-cultural experience lived by Saudi students transitioning from their home country to another. Call the experience of transitioning ‘experience X’ and call Saudi students ‘group Y’. The research sought to examine the major question and the supplementary questions around which the study revolved, which was the following: “*What does the experience X look like for the individuals belonging to group Y?*”

As the question is broad in scope and quite complex, we decided to address it from a particular angle to grasp the essence of the students’ experience rather than providing a superficial description or a personal reflection of the experience. The efforts were directed to identify the most prominent overt display of the students’ experience; the focus was on investigating the most invariant and essential aspect of their experience. From this viewpoint, the research was directed to the quest of ‘what’ individuals encounter and ‘how’ they encounter it. This aim is characterised by the research design as exploratory (e.g., Blumer, 1986; Stebbins, 2001; Ritchie and Lewis, 2003). Exploratory research design allows researchers to “taste” and experience social phenomena and provides a journey of discovery that consists of adventure (Willig, 2008) and surprise. The researcher, guided by the research inquiry, may arrive to discover an unanticipated phenomenon.

In particular, the study of cross-cultural experience involves two aspects: first is what we can call a “transitioning experience” between two cultures. The second is the potential impact that “transitioning experience” has upon the identity of those individuals who lived the experience. The conceptualisation of the phenomenon (i.e., cross-cultural experience) must be addressed, and the theoretical perspective informing its conceptualisation should be considered while developing such a phenomenological approach.

Two theoretical perspectives can allow understanding the phenomenon of cross-cultural transition: the first one is the sociocultural theory, which has been developed from Vygotsky’s works (e.g., Vygotsky, 1978; Doelling and Goldschmidt, 1981; Cole, 1995; Wertsch et al., 1995); and the second one is symbolic interactionism theory, which draws on the works of Mead, Blumer, and others (e.g., Kuhn, 1964; Mead, 1967; Blumer, 1986; Denzin, 1992; Clammer et al., 2004; Urrieta, 2007). These two perspectives informed the conceptualisation of the research phenomenon and how the phenomenon has been approached methodologically. For what concerns Vygotsky and neo-Vygotskian authors (and their sociocultural perspectives), they facilitate our understanding of the phenomenon of cross-cultural transition and its investigation. For example, Vygotsky and neo-Vygotskian authors conceptualise the individual ability to adjust to the new culture. The assumption that underlies the investigation is that individuals can acquire new cognitive developmental patterns of thought employing what these authors call “mediational assistance of tools, signs, and other cultures” (Kozulin, 2018).

For what concerns instead, the symbolic interactionism approach, this latter allows researchers to understand cross-cultural experienced phenomena by taking into consideration the role of symbolic meanings in forming individuals’ experience. The core assumption developed from this perspective is that symbolic meanings are developed, while individuals acquire their understanding of both their internal and external world.

To analyse cultural identity and this transitioning experience, another relevant aspect to consider is that symbolic interactionists assume that the definition of individual self and identity are both constructed in and played out through interaction with the environment and the other selves surrounding us. As stated by Hollander et al. (2011), the most basic requisite for symbolic interaction is the existence of social selves who come together to share information, emotions, and goods—the full range of human activities. The conceptions that people have of themselves, and others shape how they present themselves. In turn, how they present themselves allows others to infer what actors privately think of themselves and others (p. 123). Another aspect to be noted is that in the context of cross-cultural transitioning, cultural identity reflects how individuals think and feel about belonging to their culture and to the larger society from which they come from; it is in the essence of their experiences, the sense of belonging to, or attachment with, either or both cultural groupings. To fully appreciate this, we need to “borrow” from different authors’ arguments, ideas, and theoretical perspectives and adopt the hybrid perspective that we mentioned.

With this in mind, we present an overview of our research aims and the attributes that they involve: exploration and philosophical assumptions about sought knowledge.

### Exploration

The study process is not a recipe to follow but rather a journey to take, and as Willig (2008, p. 2) pointed out, the concept of research ‘has moved from a mechanical (how-to-apply-appropriate-techniques-to-the-subject-matter) to a creative (how-can-I-find-out?) mode’.

A study should be designed to maintain the subjective approach of the researcher towards the exploration of the phenomenon being investigated, as well as to appreciate the inter-subjective nature of the approach involved in the investigation of the phenomenon itself. A phenomenological qualitative method allows to track empathy and recognition of both the researcher’s and the participant’s subjectivity in relation to the phenomenon being explored.

The design is aimed to provide the researcher and the audience, with an opportunity to test and experience the phenomenon through descriptions of the essence of the experience. By concentrating on exploration as an essential aim, we evoke flexibility—the type of flexibility that allows researchers to shift between lines of inquiry and move from one activity to another to uncover the structure of the experience. The direction and proposal concerning the activities should be open enough to accommodate the complexity and ambiguity that surrounds any examined phenomenon. Flexibility consists of merging the exploratory research with phenomenological and qualitative practices.

### Philosophical Assumptions About Sought Knowledge

We consider ontological assumptions, that is, specific beliefs about some aspect of reality, and epistemological assumptions, that is, specific beliefs about some aspect of knowledge, that constitutes the phenomenon being the object of the investigation. Ontological and epistemological assumptions are considered an essential part of the research design. Therefore, researchers should identify these assumptions while engaging with the research process, as they will play a significant role in framing the research questions and justifying the research methodology, on the one hand, and the methods and activities, on the other hand (Guba, 1990; Guba and Lincoln, 1994; Crotty, 1998; Denzin and Lincoln, 2003; Creswell, 2009; Høffding and Martiny, 2016; Martiny et al., 2021; Høffding et al., 2022).

#### Ontological Assumptions of the Phenomenological Investigation

Ontological assumptions are, here, propositions about the nature of social reality—that is, what exists in a social context (Crotty, 1998; Blaikie, 2000). They relate to questions about reality: for example, what reality does exist? Does it have an external existence or is it internally constructed? However, not all phenomenologists consider ontological issues a real concern for designing and practising qualitative inquiry. That is because the ideas of phenomenology appeared as a reaction to the scientific positivist philosophical view of knowledge that dominated the philosophy of science. The phenomenological arguments, when they first appeared, were not concerned with ontological questions but rather they focussed on providing an alternative epistemological approach about how we can access knowledge that tends to be subjective and internally mediated. In other words, phenomenology, in its original form, is an attempt to explore the relationship between the knower and the known, which is an epistemological issue in philosophy rather than an ontological position. The main issue that concerns phenomenology, from these perspectives, is whether we assume or not that reality exists outside of human consciousness, i.e., before or independently of whether we think and reason about it. The epistemological question needs to be answered from both positions. The epistemological question is the real dilemma, and this concerns who is invested in the study of human consciousness. From this perspective, what is provided by human consciousness is our social reality, regardless of its internal existence, before we think about it. Knowledge is what research usually attempts to provide, therefore, it is what should concern a researcher. According to Spinelli (2005, p. 15), “We have no idea whether ‘things in themselves’ truly exist. All we can say is that, as human beings, we are biased towards interpretations that are centred upon an object-based or ‘thing-based’ world”. In addition, ontological assumptions should be identified clearly before one practice phenomenological research. This perspective has relied on Heidegger’s thesis, which moved the discussions concerning phenomenology to the ontological level when he discussed the philosophy of existence and being from a phenomenological perspective (Laverty, 2003; Tarozzi and Mortari, 2010).

The basic philosophical assumption underlying a phenomenological investigation is that truth can be found and can exist within the individual lived experience (Spiegelberg and Schuhmann, 1982). Our study is based on arguments about the existence of a social world as internally mediated, which means that as humans, we must interact with this existence and construct meanings based on our culture and beliefs, historical development, and linguistic symbols.

In our work, we considered an internal reality that was ‘built up from the perception of social actors’ (Bryman, 2008, p. 18) and that was consistent with the subjective experiences of the external world (Blanche and Durrheim, 1999). This assumption was supported by Dilthey (1979, p. 161) who said that ‘undistorted reality only exists for us in the facts of consciousness given by inner experience (, and) the analysis of these facts is the core of the human studies’.

The meanings emerged from the research methods and activities, and from this systematic interaction with the participants in this research and from sharing their experience, for example, about our work on students transitioning from their home country to the novel educational social context. These meanings should be considered a central part of the social reality that a study should report upon. This assumption underlies and merges implicitly with the second level of assumptions, the epistemological assumptions of phenomenology.

#### Epistemological Assumptions of the Phenomenological Investigation

In qualitative research, the researcher can be considered the subject who acts to know the phenomenon that is considered as the object. Accordingly, the phenomenon of cross-cultural transition between two cultures can be seen as an (object) for the deed of the investigator who is seen as (subject). Identifying the relationship between subject and object is essential to developing a coherent and sound research design. The following epistemological considerations are relevant to the current investigation.

##### Intentional Knowledge

The first element is intentionality. This concept is at the heart of the phenomenological approach (Moustakas, 1994; Crotty, 1998; Husserl and Hardy, 1999; Barnacle, 2001; Creswell et al., 2006; Tarozzi and Mortari, 2010). The original idea of phenomenology was built on this concept, introduced by Brentano (1874). Intentionality is the direction of the content of a mental state. This is a pervasive feature of many different mental states: beliefs, hopes, judgements, intentions, love, and hatred. According to Brentano, intentionality is the mark of the mental: all and only mental states exhibit intentionality. To say that a mental state has intentionality is to say that it is a mental representation and that it has content. Husserl, who was Brentano’s student, assumed that this essential property of intentionality, the directedness of mental states onto something, is not contingent upon whether some real physical target exists independently of the intentional act itself. This is regardless of whether the appearance of the thing is an appearance of the thing itself or an appearance of a mediated thing. Such consciousness and knowledge of the thing amount to perspectival understanding. Therefore, a person’s understanding is an understanding of a thing or an aspect of a thing (object). The key epistemological assumption, derived from Husserl’s concept of intentionality, is that the phenomenon is not present to itself; it is present to a conscious subject (Barnacle, 2001). Therefore, the knowledge that an individual hold about the phenomenon is mediated and one cannot have ‘pure or unmediated access’ which is other than a subjective mediated knowledge (Barnacle, 2001, p. 7). We have access only to the world that is presented to us. We have an intention to act, to know what is out there, and we only can have access to an intentional knowledge that the knower can consciously act towards (Hughes and Sharrock, 1997). Therefore, the assumption held here is that knowledge is the outcome of a conscious act towards the thing to be known (Hughes and Sharrock, 1997).

##### Subjectively Mediated Knowledge

The second epistemological assumption is related to the previous one, that of intentionality. It is that we either assume that the social world and a phenomenon do exist outside of our consciousness (see, for example, Vygotsky, 1962; Burge, 1979, 1986), or that they do not, but we are able only, as individuals, to interact with it and produce meaning for it through a conscious act. Consciousness is the ‘medium of access to whatever is given to awareness’ (Giorgi, 1997, p. 236); therefore, epistemologically, only subjective knowledge can be known about the experienced world. This assumption leads to the next epistemological assumption held in this investigation, which claims that knowing other people’s experiences is the outcome of constructed and dialogical knowledge.

##### Constructed Dialogical Knowledge

By stating that the knowledge obtained from a phenomenological study is constructed dialogically, we differentiated between philosophical knowledge on life experiences, and the knowledge provided by certain research practices that explore and understand other people’s descriptions of their lived experience (Giorgi, 2006a,b; Finlay, 2008).

From a phenomenological perspective, we assume that knowledge provided through the research activities is a result of the researcher’s and participants’ interactions with the phenomenon that is subject to the investigation. The essence of the argument here is that the ‘experience’ is best known and represented only through dialogical interaction: an interpretative methodology that analyses (spoken or written) utterances or actions for their embedded communicative significance (Linell, 2009). For what concerns us, interaction occurred between two inseparable domains: between the conscience of the researcher and the participants, and between these consciousnesses and the phenomenon explored. The qualitative methodology provided a direction for this study by way of navigating through the first domain, which was the interaction between researcher and participants. The first domain had two levels of interaction, with the first being the relationship between researcher, participants, and raw data as a dialogical relationship—a dialogical relationship in the sense that the researcher is actively engaged, through dialogue (in the form of spoken or written communicative utterances or actions), in constructing reasonable and sound meanings from the data collected from the participants (Rossman and Rallis, 2003; Steentoft, 2005). The importance of such a dialogical relationship, in phenomenological research, is supported by Rossman and Rallis (2003).

## Phenomenological Qualitative Methods and Strategies

Two forms of phenomenological methodologies can be noticed in the literature of qualitative research: descriptive phenomenology and interpretive phenomenology (Moustakas, 1994; Lopez and Willis, 2004). Descriptive phenomenology was derived mainly from the philosophical work of Husserl and particularly from the idea of transcendental phenomenology (Moustakas, 1994; Lopez and Willis, 2004; Giorgi, 2010). In contrast, an interpretive phenomenological methodology was derived from the works of scholars like Heidegger, Merleau-Ponty (1962), Gadamer (2000), and Gadamer and Linge (2008).

These approaches overlap in the research methods and activities and are used to assist the research by promoting engagement with responsive and improvised activities rather than with mechanical procedures. In fact, key principles of both descriptive and interpretative phenomenology are peoples’ subjective experiences and the meanings they ascribe to their lived world and how they relate to it (Langdridge, 2007). No definite line distinguishes or separates these two approaches or attitudes. Deploying both binaries is what differentiates our phenomenological qualitative approach from other qualitative approaches in the field (see, for example, Finlay, 2008; Langdridge, 2008).

### Descriptive Attitude

**Figure 1:**
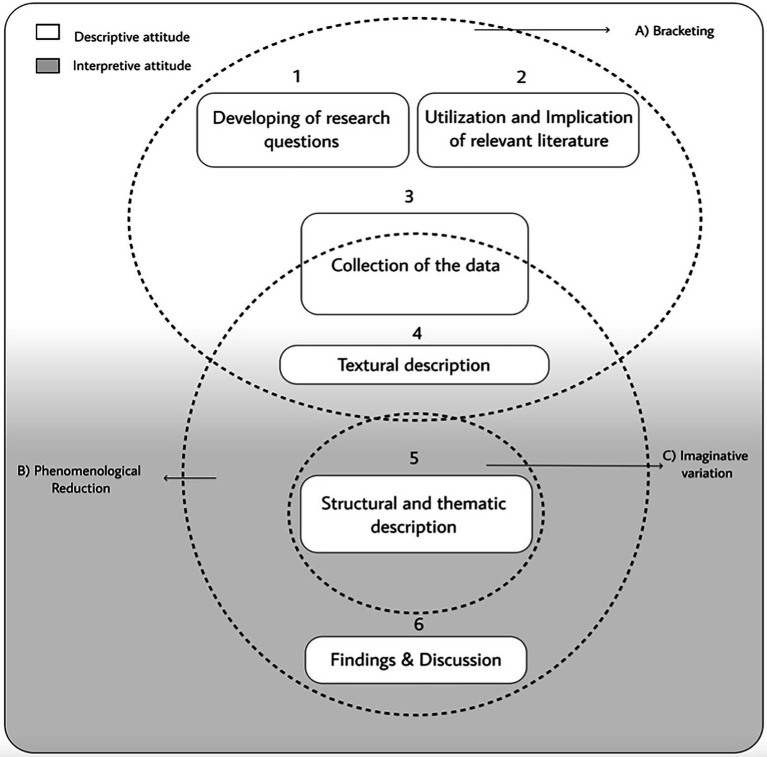
The hybrid phenomenological qualitative method.

The descriptive attitude in ‘the sense of description versus explanation’ (Langdridge, 2008, p. 1132; Ihde, 2012) occurs where the emphasis is on describing what the researcher hears, reads, and perceives when entering the participants’ description of their experience. According to Ihde (2012, p. 19) it is that attitude that consists in ‘describe phenomena phenomenologically, rather than explain them’. The whole phenomenological qualitative approach process is not description vs. interpretation, since interpretation is inevitably involved in describing and understanding the description of other people’s lived experiences (Langdridge, 2008). As presented in Figure 1, the descriptive attitude is served by the bracketing mode and by the reduction process in order to generate a textural description of the described lived experience (Moustakas, 1994; Creswell, 2007).

### Bracketing

Bracketing refers to the efforts that should be made to be open to listening to and observing the described phenomenon with fresh eyes. It is an attempt to put aside any prejudgements regarding the phenomenon being investigated (Salsberry, 1989; Moustakas, 1994; LeVasseur, 2003; also see a critical discussion in Zahavi, 2019a, 2020, 2021, and in Zahavi and Martiny, 2019). This mode also allows one to engage phenomenologically with the reduction process concerning the participants’ descriptions of their lived experiences. What the bracketing mode offers to a phenomenological qualitative study is: (1) temporary suspension of any prejudgements or assumptions related to the examined phenomenon that might have limited and restricted how the phenomenon appeared for the participants, while being aware that it is impossible to be completely free from any presuppositions; and (2) assistance in maintaining the involvement of previous experiences and perceptions about the phenomenon to recognise and realise what constitutes other aspects of the explored experience. According to Moustakas (1994, p. 85) adopting a bracketing status allows that ‘whatever or whoever appears in our consciousness is approached with an openness’. The bracketing mode influences most stages of the research activities about the following aspects:

– Forming descriptive research questions free from presuppositions to guide and direct the research enquiry, leading to the achievement of a study’s aims.
– Responding to and engaging with previous works that were concerned with the same experience.
– Conducting descriptive interviews that allow participants to share and describe their lived experiences.
– Re-describing the described experience with careful treatment of the data included, maintaining the involvement of the researcher, and avoiding being selective or discriminating in the re-description of the experience.

### Phenomenological Reduction

Phenomenological reduction is the process of re-describing and explicating meaning from the described experience (Giorgi, 1985, 2006a,b; Moustakas, 1994; Crotty, 1998; Todres, 2005; Creswell, 2007; Finlay, 2008). Such strategies are used to underlie the data analysis process. For Moustakas (1994) and others (e.g., Todres, 2005, 2007), the phenomenological reduction of human experience deals with two dimensions of the experience: texture and structure.

The texture is the ‘thickness’ of an experience (Todres, 2007, p. 47); it is a description of what the experience is like. Accordingly, the texture is an extensive description of what happened and how it appears to the researcher. The texture is the *qualitative* feature of the experience (Moustakas, 1994; Creswell, 2007). The structure of the experience deals with emergent themes, and these describe the essential aspect of the experience. Such themes ‘can be grasped only through reflection’ on the textural descriptions of the participant’s experience (Keen, 1975, as cited in Moustakas, 1994, p. 79).

### Interpretive Attitude

The interpretive attitude is the second strategy to be used to approach the data. It is part of the phenomenological approach towards discovering the essential structure and meanings of the experience as described by the participants. The interpretive attitude is part of the methodological strategies used to search for the essence of the experience. This approach is used mainly in the final stages of the research activities when the data analysis is being conducted.

As Finlay (2008, 2009) argued, ‘interpretation (in phenomenological practice) is not an additional procedure: It constitutes an inevitable and basic structure of our “being-in-the-world.” We experience a thing as something that has already been interpreted’ (p. 10). Therefore, to achieve a meaningful description and understanding of the essential aspect of an experience, we should move from the bracketing mode to an imaginative variation mode to reflect on the first step of the phenomenological reduction, which is a textural description.

### Imaginative Variation Mode

In the phenomenological literature, imaginative variation is akin to the induction process in that it aims to extract themes and essential meanings that constitute the described experiences (Klein and Westcott, 1994; Moustakas, 1994; Giorgi, 2006a,b, 2009; Creswell, 2009). It should be mentioned, however, that in the phenomenological practise, shifting from a descriptive to an interpretive attitude is ‘interpretive so far’ (Klein and Westcott, 1994, p. 141). It shall be noted that usually applying phenomenology within qualitative methods is seen as working with a version of ‘factual variation’ that, in comparison to ‘imaginative variation’, works with qualitative data (as described in Høffding and Martiny, 2016). However, since our approach is not purely fitting within the epistemological assumptions of positivism and neo-positivism, but rather it reflects the epistemological assumptions of the hermeneutical approach, we prefer to adopt ‘imaginative variation’, and remain consistent with our hybrid view that attempts to balance descriptive and interpretative methods of investigation. The imaginative variation mode enables a thematic and structural description of the ‘experience’ to be derived within the process of phenomenological reduction. This mode assists in focusing on the second aspect of the research, which requires an examination of how the experience might affect the cultural identity of the participants, that is, that part of their self-conception that is typically influenced by the cultural background of their country of origin, and that is responsible for shaping their social values and beliefs. This strategic mode can guide the researcher to shift from the descriptive to the interpretive attitude. According to Von Eckartsberg (1972, p. 166) such a mode ‘constitutes the reflective work, looking back and thinking about this experience, discovering meaningful patterns and structures, universal features that are lived out concretely in a unique fashion’. This will be considered describing “past experience” as “mediated experience” in the final analysis. And mediation is an essential process that individuals engage with in relation to their experience. Reflecting on people’s personal experiences requires mutual and reciprocal respect between researcher and participants (Klein and Westcott, 1994). This aspect allows the researcher to engage with the texture of the participant’s personal experience, to reflect on it, and to decide on possible meanings in relation to the whole context. It also allows the participants to evaluate the researcher’s reflection. This methodological mode can play a significant role in the process and activity of data analysis.

## Practising Phenomenology: Methods and Activities

We provided an overview of the methodology that we endorse as hybrid since it embeds both descriptive and interpretive phenomenological attitudes. To implement and explicate this approach in the practice of the research, we can take suggestion of Moustakas (1994) about organising the phenomenological methods around three categories: (1) methods of preparation, (2) methods of collecting data and gaining descriptions about the phenomenon, and (3) methods of analysing and searching for the meaning. These categories are useful when it comes to conducting a phenomenological qualitative study because they allow for the reporting of the most significant methods and ensure that activities are conducted in a logical order.

### Methods of Preparation, Activities, and Data Collection

If the nature of the study is emergent, like in most qualitative research (e.g., Creswell, 2009; Hays and Singh, 2011), the research purpose and questions are emergent too; they grew initially from personal experience and then emerge through the process of conceptualising a research topic around experience being investigated, for example, the experience of cross-cultural transition lived by individuals who move from their own cultural and educational context to a different one. In our past work, this was the transition of Saudi students, both males and females, from Saudi Arabia to Australia. These students experienced the transition from a gender-segregated, deeply religious cultural and educational social context to a different one, where gender-mixed interactions are not limited to members of one’s own family, such as in Saudi Arabia. In Australia, these students experienced life in a gender-mixed educational social context that is not built on religious pillars. The experience that we investigated consisted of: the cross-cultural transition to a different educational social context. As Giorgi (1985), Van Manen (1990), Moustakas (1994), and other phenomenologists have stated, aspects that are core to the interviews are the following: (1) general attributes of the conducted interviews, (2) criteria of selection for potential participants, (3) ethical considerations of dealing with human participants, and (4) the interviewing procedures and some examples.

#### Attributes of the Conducted Interviews

The main attributes of the interviews may be summarised as follows:

As an interview is influenced by the mode of bracketing, prior to each of the interviews it is necessary to elicit the participant’s experience separately from any comparison with one’s own. The interviews are about what the participants want to say rather than what the main researcher wants them to say or what the main researcher expect them to say. It is important to point out that the interviews are designed in such a way to encourage discursive answers rather than affirmative or negative answers (as discussed in Høffding and Martiny, 2016). Engaging with the interviews has the scope of seeking new views and perspectives about the phenomenon that is being investigated, and not simply to confirm or disconfirm what is already known about that phenomenon.

Here is an example of spontaneous answers to open questions taken from our previous work: Z. talks freely about the first week of experience in the novel educational social context in Australia: “Explicitly, the first class was horrible; was very bad. It is probably because I have not been in such position [mixing with males]. So, I was silent most of the time; I did not talk with any one most of the time; and I isolated myself in corner…. Mixing [with unknown males] is difficult for me because I have to deal with foreign men and I do not know them … I do not have a problem to speak with men. But the problem for me [is that] sometimes I think what if this man cross the limits between how I can deal with such behaviour. So I preferred to stay away from the men. In the first time it was hard, I could not do anything by myself. Many times, I just cried. The life [here] was mysterious in the beginning.”

And again towards the end of the stay in Australia, Z. spontaneously shares how her worldviews about herself have been changed by being in a gender-mixed educational environment. For example, Z. stated clearly that she is now confident ‘to deal with male’—after all the ‘scariness’ and ‘horribleness’ that was felt in the beginning. She learned from her experience in a gender-mixed environment how to make her own rules that males cannot cross. Z. said: “… Being here has changed my personality completely…. The most important advantages from (being here) refined my personality in a good way, and I became more independent…. I refined my personality. Not only me, who realised that, but my family also said that: Z. has changed…. Finally, I learned how to deal with man with confidence and how to make my own rule. So When I come back to Saudi Arabia, I will be more confident.”

During the interviewing activity, is also important to share experiences with the interviewees in order to practice empathy (Corbin and Morse, 2003; Dickson-Swift et al., 2006; August and Tuten, 2008; Mitchell and Irvine, 2008; Mallozzi, 2009) and be respectful for what they feel about their experiences (Klein and Westcott, 1994). These techniques are outlined to show interviewees that the researcher is interested in hearing detailed accounts (Hays and Singh, 2011) about their experiences. As Hays and Singh (2011) have suggested, such involvement during an interview activity may encourage participants to share their experiences more freely, if they feel they are in a friendly situation. The advantages of this technique can be reflected in the descriptions of the answers provided and in the participants’ helpfulness in reviewing the transcribed interviews and adding or correcting data.

#### Selection of the Participants

A purposive sampling method can be used to select the participants. This is a type of nonprobability sample. The main objective of a purposive sample is to produce a sample that can be logically assumed to be representative of the population. This is often accomplished by applying expert knowledge of the population to select in a nonrandom manner a sample of elements that represents a cross-section of the population. For example, in our past work, such expertise was given by the author being a Saudi citizen who went to study in Australia. Such methods are considered fitting for most investigations if one wants ‘to discover, understand, and gain insight … from which the most can be learned’. Another reason to use a purposive sampling method is that in qualitative, particularly in phenomenological inquiry, the aim is not to generalise findings to a population but to develop insights and in-depth exploration of an under-researched phenomenon (Onwuegbuzie and Leech, 2007). The concern is not about the number of participants. Rather, the focus should be on the intensity of participation and the diversity of the participants. Moustakas (1994) suggested that the number of participants in a phenomenological study can be from 1 to 20, depending on the time frame (see, Halldórsdóttir, 2000; Morse, 2000; Starks and Trinidad, 2007; Jones and Lavallee, 2009).

This section describes how the data and reports on the activity conducted can be treated to generate findings from the interviews. The following series of processes is indicative of the path followed to arrive at the findings for this research, which relied heavily on the works of Hycner (1985), Moustakas (1994), Giorgi (1997), and Wertz (2005) when a plan for data treatment is developed. Warning of Hycner (1985) against using the term data analysis when engaging in a phenomenological approach has been considered. The concept of analysis involves breaking things into parts, while phenomenology is about potting parts of any experience (phenomenon) together to get a sense of the whole, to get into phenomenological “reduction.” We are looking for “the essence.” This requires getting a sense of the whole rather than of the part. Therefore, we prefer to use “explication.” Explication usually points to the process of being explicit about the constituents of the whole phenomenon. Using a popular term like analysis may be inconsistent with how the data are treated because the term analysis usually implies a process of breaking things into parts. Therefore, to avoid misleading uses of terminology, the suggestion is to use the term data explication, which Groenewald (2004) suggested. Explication usually points to the process of being explicit about the constituents of the whole phenomenon (Hycner, 1985; Groenewald, 2004).

#### The Interviewing Procedure

In order to capture and explicate the essence and the structures constituting the experience encountered by the participants nine steps can be followed: (a) transcribing participants’ interviews, (b) developing a sense of the whole, (c) developing meaning units for each participant’s experience (horizontalisation), (d) clustering relevant units of meanings, (e) translating the meaning units, (f) developing textural (i.e., narrative) descriptions for each individual, (g) searching for essential structures that could express the entire textural description, (h) evaluating the textural description, and (i) synthesising the structure from all participants’ accounts. Each step is addressed in further detail in the remainder of the paper.

#### Transcription

After the interviews are conducted with all the participants, the interview recordings are transcribed. After having confirmed the privacy and confidentiality statements that are provided by the third-party transcribers are confirmed, verbally and by email, interviews are sent to the transcribers, and records should be deleted after the completion of the transcription process.

#### Developing a Sense of the Whole

Following the transcription process, the second step consists in developing a general sense for each participant’s description. This involves listening to all the recordings several times as well as reading the transcripts several times. Repeating the procedure is useful to make sure the content of the interviews is carefully approached: In fact, this process helps the investigator to become familiar with the context of the units of meaning and themes that they sought to extract in the next step. At this stage, the goal is to get a general sense of what participants had told the investigator about their experience. This sense provides a foundation for the following process of data explication. Engaging in this activity helps the investigator to switch on and keep the focus on the phenomenon itself, which appear within the descriptions of the participants.

It is essential to the phenomenological attitude to pay full attention to both the spoken and written forms of the data. Developing a sense of the wholeness and of the entirety of what everyone had expressed regarding their experience is necessary because the goal of the investigation is to find the essential meanings of the experience as encountered by the participants (Hycner, 1985; Moustakas, 1994). Each transcript and record should be read and listened to separately and at different times. This step allows getting an overall sense of the data.

#### Developing Meaning Units for Each Participant’s Experience (Horizontalisation)

After transcribing the interviews, and once a general sense of the whole description of the phenomenon has been gained, it is possible to formally engage with the data treatment in order to extract the invariant meaning units and themes that constitute the experience encountered by the participants. Every statement, phrase, sentence, and paragraph in each transcript is examined to elicit statements relevant to the experience. At this stage, the attitude is to go through the transcripts with an open-minded attitude, as much as possible (Hycner, 1985). This means to stay in the bracketing mode and be as descriptive as possible. Moustakas (1994) called this stage of data treatment ‘horizontalisation’, as this is where the descriptions of each individual turn to a horizon. The horizon, in the discussion of the phenomenological data treatment, refers to the context from which an experienced phenomenon could appear; it is the source that comprises the core themes and meanings of the experienced phenomenon. The notion of phenomenological ‘horizon’ has been conceptualised differently according to which philosophical perspective is adopted. For example, the term can appear in Nietzsche, Husserl, and Heidegger, wherein it has been used to refer to very different concepts (Scott, 1988; Von Eckartsberg, 1989; Husserl and Hardy, 1999; Heidegger and Dahlstrom, 2005; Christofi and Thompson, 2007). Therefore, to avoid confusion around the term ‘horizon’, the term is presently substituted by the expression ‘meaning units’, as this term refers directly to what is being achieved at this stage of data explication. Invariant meaning units are the non-repetitive or overlapping statements that explicitly or implicitly capture a moment, or several moments, of what has been experienced (i.e., the texture of the experience). To develop the meaning units from the participants’ accounts, the following sub-steps come next: *listing all statements relevant to the experience*, and *going through the list of statements* by checking each statement against two criteria suggested by Moustakas (1994, p. 121): (1) Is the statement essential for understanding the phenomenon being studied? (2) Can it be abstracted and labelled? Any statement that conforms to these criteria was included as an invariant meaning unit. The statements that did not meet these criteria—those that are repetitive, overlapping, or unclear—are eliminated.

This process is difficult as well as the most critical one (Wertz, 1985) because the entire investigation depends on these units of meaning. It takes time to be confident in eliminating some statements that do not meet the relevancy requirement.

#### Clustering Relevant Units of Meaning Into Groups

After developing the list of relevant meaning units, it is necessary to go through them several times in the mode of imaginative variation to identify a significant theme that could be clustered as a possible unit of meaning. Turning the attention to imaginative variation is useful in examining identified meaning units reflectively, adding the dimension that allows subjective judgements. To avoid inappropriate subjective judgements, it is important to keep bracketing one’s own presuppositions to see what might possibly emerge (Von Eckartsberg, 1972; Moustakas, 1994). However, it should be acknowledged that the researcher’s prior experience cannot be completely isolated, as the researcher must use their constituted mind (Al-Jabri, 2011b) to understand and to identify the emerging themes. To minimise this necessary risk, it is recommended to ask external reviewers to be independent judges and check for consistency under the themes that are selected. At this stage, each case is still being treated individually to identify the unique experience of each participant. This approach is also useful for obtaining an in-depth understanding of the data, rather than rushing into the whole. These clusters are the core themes to use in organising the invariant meaning units (here referred to as “the core themes of the experience” of the phenomenon; Moustakas, 1994, p. 121), before revisiting them to develop the textural description of the participant’s experience. This step helps organise the textural description of the experience (Moustakas, 1994).

#### Translating the Meaning Units

In previous stages, the data explication can be kept, as much as possible, to what is expressed by the participants. This should all be done in the primary language spoken by the participants (i.e., native language or most used language, since native language is not always the best know language—especially in individuals who grew up or were educated in a language other than the language of the family of origin) to allow participants to express their experience by using their ‘tools’ (Vygotsky, 1962). This is important for getting a deeper description of the experience because language interacts with thinking and consciousness dialectically. The underlying assumption is that language, as a mediating tool, shapes participants’ experience, and it is also a result of experience, and a significant constituent of the epistemological system of a given cultural group. Furthermore, like Burkitt (2011, p. 269), we maintain that sociocultural theory and symbolic interactionism theory promote an assumption ‘that language does not express thoughts that already exist but provides the tools to bring thoughts into existence’.

In our previous work, the preferred language during the interviews was Arabic, spoken both by the researcher and the participants. Subsequently, the interviews were translated into English to be accessible to the scientific community internationally.

#### Developing a Textural Description for Each Individual

The sixth step consists in constructing a description of the texture of the experience from the clustered meaning units. This step provides rich, thick descriptions of each individual’s experience. The textural description, which is by now translated in the language in which the study is conducted (if different from the language in which the participants expressed themselves during the interviews), presents what is experienced by each participant to provide this thick description, it is important to ask the following question for every invariant meaning unit: what can possibly appear as the texture of the participant’s experience?

It should be indicated that as part of the process at this stage, some of the texture can appear in different meaning units, which means there is still some repetition and/or overlapping of the meaning units that are not eliminated in the fourth step.

#### Searching for Essential Structures That Could Express the Entire Textural Description

After constructing textural descriptions for each participant, it is time to deploy the imaginative variation mode again to search for essential structures that could encompass the entire textural description of the participant: a possible theme that could be the essential structure of the experience of this participant—essential in the sense that the experience could not be described without this theme, or themes. At this stage, the interpretive attitude comes into play to help the investigator to identify the structure of the textual description. The interpretive attitude is important during this process because it involves deep contemplation and reflection on the textural description to capture the structural meaning.

#### Evaluating the Textural Description and Structural Theme of Each Participant’s Experience

Once the textural and structural descriptions are ready, we have reached the evaluation step. In this step, we suggest adopting the following criteria from phenomenological guidelines of Hycner (1985): Do the participants agree with the identified textures and structures to represent what they had described in the interview? Did the investigator miss any other essential aspect of the participants’ experiences that the participants would like to add?

#### Synthesising the Structures From All the Participants’ Accounts

The final step consists in synthesising the structures of the material gathered from all participants’ accounts to ‘communicate the most general meaning of the phenomenon (Giorgi, 1985, p. 20). Because this activity is the final activity in terms of the data treatment, the main research question of the study must be addressed directly.

The discussion over the structures that emerge from all participants’ interviews should take the form of writing a composite summary to describe how the experienced phenomenon is seen by the participants (Giorgi, 1985; Hycner, 1985; Van Manen, 1990; Moustakas, 1994). In this summary, it is important to concentrate on the common aspects of the experience as an essence of the phenomenon. At the same time, it is crucial not to ignore the unique and different views of the participants.

## Conclusion

In this article, we have presented a hybrid phenomenological method embedded in qualitative analysis that we suggest should be deployed in educational research. Our analysis is relevant to those researchers interested in doing qualitative research and in those interested in adapting phenomenological investigation to understand experiences in different educational groups and social contexts, such as cross-cultural transitions, as we have shown. A phenomenological qualitative method provides a theoretical tool for educational research as it allows researchers to engage in flexible activities that can describe and help to understand complex phenomena, such as various aspects of human social experience.

## Author Contributions

All authors listed have made a substantial, direct, and intellectual contribution to the work and approved it for publication.

## Conflict of Interest

The authors declare that the research was conducted in the absence of any commercial or financial relationships that could be construed as a potential conflict of interest.

## Publisher’s Note

All claims expressed in this article are solely those of the authors and do not necessarily represent those of their affiliated organizations, or those of the publisher, the editors and the reviewers. Any product that may be evaluated in this article, or claim that may be made by its manufacturer, is not guaranteed or endorsed by the publisher.

## References

[ref1] Alhazmi A. A. Nyland B. (2013). The Saudi Arabian international student experience: from a gender-segregated society to studying in a mixed-gender environment. Compare 43, 346–365. doi: 10.1080/03057925.2012.722347

[ref2] Alhazmi A. A. Nyland B. (2015). Contextualization of Saudi international students’ experience in facing the challenge of moving to mixed gender environments. Am. Int. J. Contemp. Res. 5, 87–97.

[ref3] Al-Jabri M. A. (2011b). The Formation of Arab Reason: Text, Tradition and the Construction of Modernity in the Arab World. London: I. B. Tauris.

[ref4] August R. A. Tuten T. L. (2008). Integrity in qualitative research: preparing ourselves, preparing our students. Teach. Learn. 22:82.

[ref5] Barnacle R. (2001). “Phenomenology and wonder,” in Phenomenology. ed. Barnacle R. (Melbourne: RMIT University Press), 3–15.

[ref6] Blaikie N. W. H. (2000). Designing Social Research: The Logic of Anticipation. Malden, MA: Polity Press, Cambridge.

[ref7] Blanche M. T. Durrheim K. (1999). “Histories of the present: social science research in context,” in Research in Practice: Applied Methods for the Social Sciences. eds. Blanche M. T. Durrheim K. Painter D. (Cape Town, South Africa: University of Cape Town Press), 1–17.

[ref8] Blumer H. (1986). Symbolic Interactionism: Perspective and Method. Berkeley, CA: University of California Press.

[ref301] Brentano F. (1874). Psychology From an Empirical Standpoint.

[ref9] Bryman A. (2008). Social Research Methods. New York: Oxford University Press.

[ref10] Burge T. (1979). Individualism and the mental. Midwest Stud. Phil. 4, 73–121. doi: 10.1111/j.1475-4975.1979.tb00374.x

[ref11] Burge T. (1986). Individualism and psychology. Philos. Rev. 95, 3–45. doi: 10.2307/2185131

[ref302] Burkitt I. (2011). “Identity construction in sociohistorical context,” in Handbook of Identity Theory and Research (New York, NY: Springer), 267–283.

[ref12] Christofi V. Thompson C. L. (2007). You cannot go home again: a phenomenological investigation of returning to the sojourn country after studying abroad. J. Couns. Dev. 85, 53–63. doi: 10.1002/j.1556-6678.2007.tb00444.x

[ref13] Clammer J. Poirier S. Schwimmer E. (eds.) (2004). Figured Worlds: Ontological Obstacles in Intercultural Relations. University of Toronto Press.

[ref14] Cole M. (1995). Culture and cognitive development: from cross-cultural research to creating systems of cultural mediation. Cult. Psychol. 1, 25–54. doi: 10.1177/1354067X9511003

[ref15] Corbin J. Morse J. M. (2003). The unstructured interactive interview: issues of reciprocity and risks when dealing with sensitive topics. Qual. Inq. 9, 335–354. doi: 10.1177/1077800403009003001

[ref16] Creswell J. W. (2007). Qualitative Inquiry and Research Design: Choosing among Five Approaches. Thousand Oaks, CA: Sage.

[ref17] Creswell J. W. (2009). Research Design: Qualitative, Quantitative, and Mixed Methods Approach. Thousand Oaks, CA: Sage.

[ref305] Creswell J. W. Shope R. Plano Clark V. L. Green D. O. (2006). How interpretive qualitative research extends mixed methods research. Res. Sch. 13, 1–11.

[ref304] Cresswell T. (2008). The Production of Mobilities. Routledge, 337–345.

[ref18] Crotty M. (1998). The Foundations of Social Research: Meaning and Perspective in the Research Process. Thousand Oaks, CA: Sage.

[ref19] Denzin N. K. (1992). The conversation. Symb. Interact. 15, 135–150. doi: 10.1525/si.1992.15.2.135

[ref20] Denzin N. K. Lincoln Y. S. (2003). Strategies of Qualitative Inquiry. 2nd Edn. Thousand Oaks, CA: Sage.

[ref21] Dickson-Swift V. James E. L. Kippen S. Liamputtong P. (2006). Blurring boundaries in qualitative health research on sensitive topics. Qual. Health Res. 16, 853–871. doi: 10.1177/1049732306287526, PMID: 16760540

[ref22] Dilthey W. (1979). Dilthey Selected Writings. ed. Rickman H. P. (Cambridge: Cambridge University Press).

[ref23] Doelling I. Goldschmidt M. (1981). *The Theory of Social/Historical Forms of Individuality Its Development State and Its Methodological Meaning for Marxist Cultural Theory/Cultural History*. Leipzig, Germany: Central Institute for Youth Research.

[ref25] Finlay L. (2006). Dancing between embodied empathy and phenomenological reflection. Indo-Pacific J. Phenomenol. 6, 1–11. doi: 10.1080/20797222.2006.11433930

[ref26] Finlay L. (2008). A dance between the reduction and reflexivity: explicating the ‘phenomenological psychological attitude’. J. Phenomenol. Psychol. 39, 1–32.

[ref27] Finlay L. (2009). Debating phenomenological research methods. Phenomenol. Pract. 3, 6–25. doi: 10.29173/pandpr19818

[ref28] Gadamer H.-G. (2000). Subjectivity and intersubjectivity, subject and person. Cont. Philos. Rev. 33, 275–287. doi: 10.1023/A:1010086224341

[ref29] Gadamer H.-G. Linge D. E. (2008). Philosophical Hermeneutics. Berkeley, CA: University of California Press.

[ref306] Gasparyan G. (2021). Double-sided transformations of culture-bound constituents in William Saroyan’s cross-cultural domain. Transl. Stud. Theor. Pract. 1, 31–44. doi: 10.46991/TSTP/2021.1.2.031

[ref30] Giorgi A. (ed.) (1985). Phenomenology and Psychological Research. Pittsburgh, PA: Duquesne University Press.

[ref31] Giorgi A. (1997). The theory, practice, and evaluation of the phenomenological method as a qualitative research procedure. J. Phenomenol. Psychol. 28, 235–260. doi: 10.1163/156916297X00103

[ref32] Giorgi A. (2006a). Concerning variations in the application of the phenomenological method. Humanist. Psychol. 34, 305–319. doi: 10.1207/s15473333thp3404_2

[ref33] Giorgi A. (2006b). Difficulties encountered in the application of the phenomenological method in the social sciences. Anal. Psicol. 24, 353–361. doi: 10.14417/ap.175

[ref34] Giorgi A. (2009). The Descriptive Phenomenological Method in Psychology: A Modified Husserlian Approach. Pittsburgh, PA: Duquesne University Press.

[ref307] Giorgi A. (2010). “Phenomenology and the practice of science,” in Existential Analysis: Journal of the Society for Existential Analysis. *Vol*. 21.

[ref35] Groenewald T. (2004). A phenomenological research design illustrated. Int J Qual Methods 3, 42–55. doi: 10.1177/160940690400300104

[ref36] Guba E. G. (1990). The Paradigm Dialog. Thousand Oaks, CA: Sage Publications.

[ref37] Guba E. G. Lincoln Y. S. (eds.) (1994). “Competing paradigms in qualitative research,” in Handbook of Qualitative Research. *Vol*. 2. Thousand Oaks, CA: Sage, 163–194.

[ref38] Halldórsdóttir S. (2000). “The Vancouver school of doing phenomenology,” in Qualitative Research Methods in the Service of Health. eds. Fridlund B. Hildingh C. (Studentlitteratur: Lund), 47–84.

[ref39] Hays D. G. Singh A. A. (2011). Qualitative Inquiry in Clinical and Educational Settings. New York, NY: Guilford Press.

[ref40] Heidegger M. Dahlstrom D. O. (2005). Introduction to Phenomenological Research. Bloomington, IN: Indiana University Press.

[ref41] Høffding S. Martiny K. M. (2016). Framing a phenomenological interview: what, why and how. Phenomenol. Cogn. Sci. 15, 539–564. doi: 10.1007/s11097-015-9433-z

[ref42] Høffding S. Martiny K. Roepstorff A. (2022). Can we trust the phenomenological interview? Metaphysical, epistemological, and methodological objections. Phenomenol. Cogn. Sci. 21, 33–51. doi: 10.1007/s11097-021-09744-z

[ref43] Hollander J. A. Renfrow D. G. Howard J. A. (2011). Gendered Situations, Gendered Selves: A Gender Lens on Social Psychology. 2nd *Edn.* Rowman & Littlefield.

[ref308] Hourigan R. M. Edgar S. N. (2020). “The foundations of phenomenology: epistemology, methodology, and analysis,” in Approaches to Qualitative Research: An Oxford Handbook of Qualitative Research in American Music Education, *Vol*. 1 110.

[ref44] Hughes J. A. Sharrock W. W. (1997). The Philosophy of Social Research. Upper Saddle River, NJ: Pearson Education.

[ref309] Husserl E. (1931). Ideas: General Introduction to Pure Phenomenology [Trans. by W. R. B. Gibson]. Macmillan.

[ref45] Husserl E. Hardy L. (1999). The Idea of Phenomenology. *Vol*. 8. Dordrecht: Kluwer Academic.

[ref46] Hycner R. H. (1985). Some guidelines for the phenomenological analysis of interview data. Hum. Stud. 8, 279–303. doi: 10.1007/BF00142995

[ref47] Ihde D. (2012). Experimental Phenomenology: Multistabilities. Albany, NY: State University of New York.

[ref49] Jarvis P. (1987). Meaningful and meaningless experience: towards an analysis of learning from life. Adult Educ. Q. 37, 164–172. doi: 10.1177/0001848187037003004

[ref50] Jones M. I. Lavallee D. (2009). Exploring perceived life skills development and participation in sport. Qual. Res. Sport Exercise 1, 36–50. doi: 10.1080/19398440802567931

[ref51] Karpov Y. V. Haywood H. C. (1998). Two ways to elaborate Vygotsky's concept of mediation. Am. Psychol. 53, 27–36. doi: 10.1037/0003-066X.53.1.27

[ref310] Keen E. (1975). A Primer in Phenomenological Psychology.

[ref52] Klein P. Westcott M. R. (1994). The changing character of phenomenological psychology. Can. Psychol. 35, 133–158. doi: 10.1037/0708-5591.35.2.133

[ref311] Koopmans L. (2015). Individual Work Performance Questionnaire Instruction Manual. Amsterdam, NL: TNO Innovation for Life – VU University Medical Center.

[ref53] Kozulin A. (2018). “Mediation and internalization,” in The Routledge Handbook of Sociocultural Second Language Development. eds. Lantolf J. Poehner M. E. Swain M. (New York: Routledge), 487–504.

[ref54] Kuhn T. (1964). “A function for thought experiments,” in The Essential Tension: Selected Studies in Scientific Tradition and Change (University of Chicago Press), 240–265.

[ref55] Langdridge D. (2007). Phenomenological Psychology: Theory, Research and Method. Pearson Prentice Hall.

[ref56] Langdridge D. (2008). Phenomenology and critical social psychology: directions and debates in theory and research. Soc. Personal. Psychol. Compass 2, 1126–1142. doi: 10.1111/j.1751-9004.2008.00114.x

[ref312] Laverty S. M. (2003). Hermeneutic phenomenology and phenomenology: a comparison of historical and methodological considerations. Int. J. Qual. 2, 21–35. doi: 10.1177/160940690300200303

[ref313] LeVasseur J. J. (2003). The problem of bracketing in phenomenology. Qual. Health Res. 13, 408–420. doi: 10.1177/1049732302250337, PMID: 12669340

[ref57] Linell P. (2009). Rethinking Language, Mind, and World Dialogically. Charlotte, NC: Information Age Publishing.

[ref58] Lodico M. Spaulding D. Voegtle K. (2006). Methods in Educational Research: From Theory to Practice. San Francisco, CA: Jossey-Bass.

[ref59] Lopez K. Willis D. (2004). Descriptive versus interpretive phenomenology: their contributions to nursing knowledge. Qual. Health Res. 14, 726–735. doi: 10.1177/1049732304263638, PMID: 15107174

[ref60] Mallozzi C. A. (2009). Voicing the interview: a researcher’s exploration on a platform of empathy. Qual. Inq. 15, 1042–1060. doi: 10.1177/1077800409334227

[ref61] Martiny K. M. Toro J. Høffding S. (2021). Framing a phenomenological mixed method: from inspiration to guidance. Front. Psychol. 12:602081. doi: 10.3389/fpsyg.2021.602081, PMID: 33746828 PMC7966507

[ref63] Mead G. H. (1967). Mind, Self and Society. Chicago University Press.

[ref64] Merleau-Ponty M. (1962). Phenomenology of Perception. London: Routledge.

[ref65] Mitchell W. Irvine A. (2008). I’m okay, you’re okay? Reflections on the well-being and ethical requirements of researchers and research participants in conducting qualitative fieldwork interviews. Int J Qual Methods 7, 31–44. doi: 10.1177/160940690800700403

[ref66] Morse J. M. (2000). Editorial: determining sample size. Qual. Health Res. 10, 3–5. doi: 10.1177/104973200129118183

[ref67] Moustakas C. (1994). Phenomenological Research Methods. Thousand Oaks, CA: Sage.

[ref68] Onwuegbuzie A. J. Leech N. L. (2007). Sampling designs in qualitative research: making the sampling process more public. Qualit. Rep. 12, 238–255. doi: 10.46743/2160-3715/2007.1636

[ref314] Polkinghorne D. E. (1989). “Phenomenological research methods,” in Existential-Phenomenological Perspectives in Psychology (Boston, MA: Springer), 41–60.

[ref69] Ritchie J. Lewis J. (2003). Qualitative Research Practice: A Guide for Social Science Students and Researchers. Thousand Oaks, CA: Sage.

[ref70] Rossman G. B. Rallis S. F. (2003). Learning in the Field: An Introduction to Qualitative Research. Thousand Oaks, CA: Sage Publications.

[ref71] Salsberry P. J. (1989). Dialogue on a research issue: phenomenological research in nursing—response. Nurs. Sci. Q. 2, 9–13. doi: 10.1177/089431848900200106, PMID: 2927793

[ref72] Scott T. (1988). “The horizon of time and ontological difference,” in Hermeneutical Phenomenology: Lectures and Essays. ed. Kockelmans J. J. (Lanham, MD: University Press of America), 67–80.

[ref73] Spiegelberg H. Schuhmann K. (1982). The Phenomenological Movement: A Historical Introduction. The Hague: M. Nijhoff.

[ref315] Spinelli E. (2005). The Interpreted World: An Introduction to Phenomenological Psychology. Sage.

[ref74] Starks H. Trinidad S. B. (2007). Choose your method—a comparison of phenomenology, discourse analysis, and grounded theory. Qual. Health Res. 17, 1372–1380. doi: 10.1177/1049732307307031, PMID: 18000076

[ref75] Stebbins R. A. (2001). Exploratory Research in the Social Sciences. Thousand Oaks, CA: Sage.

[ref76] Steentoft D. (2005). “Research as an act of learning: exploring student backgrounds through dialogue with research participants.” in *Proceedings of CERME 4*, ed. M. Bosch; February 17-21, 2005; Sant Féliu de Gixols.

[ref78] Tarozzi M. Mortari L. (2010). Phenomenology and Human Science Research Today. Bucharest, Hungary: Zeta Books.

[ref80] Todres L. (2005). “Clarifying the lifeworld: descriptive phenomenology,” in Qualitative Research in Health Care. ed. Holloway I. (Maidenhead: Open University Press), 104–124.

[ref81] Todres L. (2007). Embodied Enquiry: Phenomenological Touchstones for Research, Psychotherapy and Spirituality. London, England: Palgrave Macmillan.

[ref82] Urrieta L. (2007). Figured worlds and education: an introduction to the special issue. Urban Rev. 39, 107–116. doi: 10.1007/s11256-007-0051-0

[ref83] Van Manen M. (1990). Researching Lived Experience: Human Science for an Action Sensitive Pedagogy. New York: State University of New York Press.

[ref84] Von Eckartsberg R. (1972). Experiential psychology: a descriptive protocol and a reflection. J. Phenomenol. Psychol. 2, 161–171. doi: 10.1163/156916272X00100

[ref85] Von Eckartsberg R. (1989). The unfolding meaning of intentionality and horizon in phenomenology. Humanist. Psychol. 17, 146–160. doi: 10.1080/08873267.1989.9976848

[ref86] Vygotsky L. S. (1962). Language and Thought. Ontario: Massachusetts Institute of Technology Press.

[ref316] Vygotsky L. S. (1978). Mind in Society: Development of Higher Psychological Processes. Harvard University Press.

[ref87] Wertsch J. V. del Río P. Alvarez A. (eds.) (1995). Sociocultural Studies of Mind. Cambridge University Press.

[ref88] Wertz F. J. (1985). “Method and findings in a phenomenological psychological study of a complex life-event: being criminally victimized,” in Phenomenology and Psychological Research. 155–216.

[ref89] Wertz F. J. (2005). Phenomenological research methods for counseling psychology. J. Couns. Psychol. 52, 167–177. doi: 10.1037/0022-0167.52.2.167

[ref90] Willig C. (2008). Introducing Qualitative Research in Psychology. New York, NY: McGraw Hill/Open University Press.

[ref91] Zahavi D. (2019a). Getting it quite wrong: van Manen and smith on phenomenology. Qual. Health Res. 29, 900–907. doi: 10.1177/1049732318817547, PMID: 30565516

[ref318] Zahavi D. (2019b). Second-person engagement, self-alienation, and group-identification. Topoi 38, 251–260. doi: 10.1007/s11245-016-9444-6

[ref92] Zahavi D. (2020). The practice of phenomenology: the case of max van Manen. Nurs. Philos. 21, 1–9. doi: 10.1111/nup.12276 31441216

[ref93] Zahavi D. (2021). Applied phenomenology: why it is safe to ignore the epoché. Cont. Philos. Rev. 54, 259–273. doi: 10.1007/s11007-019-09463-y

[ref94] Zahavi D. Martiny K. M. M. (2019). Phenomenology in nursing studies: new perspectives. Int. J. Nurs. Stud. 93, 155–162. doi: 10.1016/j.ijnurstu.2019.01.014, PMID: 30795899

